# Influence of Mental Workload on the Performance of Anesthesiologists during Induction of General Anesthesia: A Patient Simulator Study

**DOI:** 10.1155/2016/1058750

**Published:** 2016-04-11

**Authors:** Hitoshi Sato, Tetsuya Miyashita, Hiromasa Kawakami, Yusuke Nagamine, Shunsuke Takaki, Takahisa Goto

**Affiliations:** Department of Anesthesiology, Yokohama City University Hospital, 3-9 Fukuura, Kanazawa, Yokohama 244-0816, Japan

## Abstract

The aim of this study was to reveal the effect of anesthesiologist's mental workload during induction of general anesthesia. Twenty-two participants were categorized into anesthesiology residents (RA group, *n* = 13) and board certified anesthesiologists (CA group, *n* = 9). Subjects participated in three simulated scenarios (scenario A: baseline, scenario B: simple addition tasks, and scenario C: combination of simple addition tasks and treatment of unexpected arrhythmia). We used simple two-digit integer additions every 5 seconds as a secondary task. Four kinds of key actions were also evaluated in each scenario. In scenario C, the correct answer rate was significantly higher in the CA versus the RA group (RA: 0.370 ± 0.050 versus CA: 0.736 ± 0.051, *p* < 0.01, 95% CI −0.518 to −0.215) as was the score of key actions (RA: 2.7 ± 1.3 versus CA: 4.0 ± 0.00, *p* = 0.005). In a serious clinical situation, anesthesiologists might not be able to adequately perform both the primary and secondary tasks. This tendency is more apparent in young anesthesiologists.

## 1. Introduction

Anesthesiologists have to perform many procedures during the induction of general anesthesia, such as drug administration and endotracheal intubation, while paying attention to the patient's vital signs at the same time. Several studies among airplane pilots and car drivers reported that performance decreased when multiple tasks were simultaneously performed [1–5]. Furthermore, recent studies reported that a higher mental workload reduced the performance of anesthesiologists, which might be a risk to patient safety [6, 7].

In the field of psychology, mental workload was assessed using several different methods [1, 2, 8–10]. However, what effects both the primary task and the additional mental workload of the secondary task would have on the performance of anesthesiologists was unknown. In this study, we assessed mental workload by measuring the capacity of anesthesiologists to simultaneously deal with primary (induction of general anesthesia) and secondary (simple mental arithmetic) tasks [11]. If performance of the secondary task is impaired, we assume that this indicates that the mental workload of the anesthesiologist is close to capacity [12, 13]. It is also to be expected that anesthesiologist performance may be influenced by level of experience. Although some studies have measured mental workloads of anesthesiologists in a clinical setting, we believed we could measure them more accurately in a simulation setting [14–16].

We hypothesized that the capacity of anesthesiologists to deal with mental workloads would differ based on experience and that it would be able to be evaluated by means of a “secondary task” because if anesthesiologists have to deal with a secondary task during the induction of general anesthesia, it will likely result in errors and poor performance in routine practice. Further, the results of the secondary task itself would be different based on the level of experience of the anesthesiologist. It was demonstrated that mental workload of the type employed here (40 math questions) produced a significant effect on the performance of both groups.

## 2. Methods

**Figure 1:**
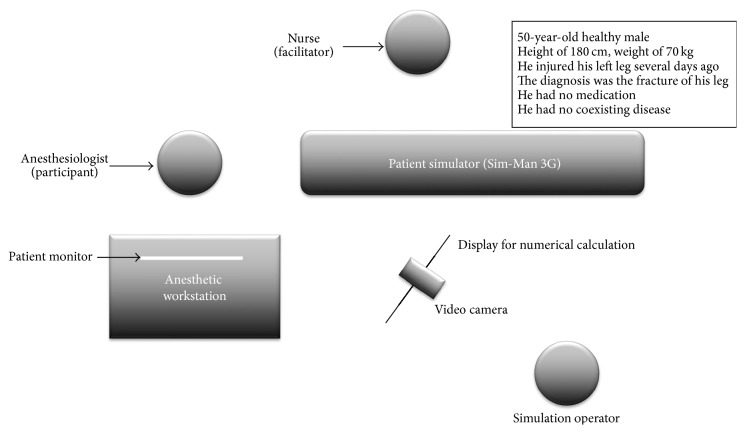
Simulation setting. A high-fidelity patient simulator (Sim-Man 3G) was set up in the operating room, and a Dräger Fabius GS*™* anesthetic workstation was used. A display for the numerical addition questions and a video camera for recording were also prepared.

Ethics committee approval was obtained from the clinical research Ethics Committee of Yokohama City University. Twenty-two participants (13 anesthesiology residents (RA group) and 9 board certified anesthesiologists (CA group)) from Yokohama City University Hospital participated in this study. We set up a high-fidelity patient simulator in the operating room (Figure 1). The characteristics of the simulated patient were as follows: A 50-year-old healthy male; height of 180 cm; weight of 70 kg; with a history of injury to his left leg several days ago, for which he was diagnosed with fracture of the leg. He was not on any medication and had no coexisting diseases.

Sim-Man 3G and corresponding software (Laerdal Medical, Stavanger, Norway) were used in this simulated study. An experienced anesthesiologist operated on the patient simulator. Participants could use the Dräger Fabius GS anesthetic workstation and ask a nurse to assist in any procedure, including the administration of any drug.

### 2.1. Scenario A: Baseline

We simulated a situation of induction of general anesthesia in healthy patients. The facilitator observed what each participant routinely did in the situation of induction of general anesthesia. We included adequate mask ventilation (key action 1), administration of inhalation anesthetics (key action 2), muscle relaxant (key action 3), and tracheal intubation (key action 4) as the key actions of induction of general anesthesia. We used this scenario as the baseline for comparison with the scores of the key actions in scenarios B and C.

### 2.2. Scenario B: Simple Addition

We simulated a situation of induction of general anesthesia in healthy patients, as in scenario A. However, before the scenario commenced, the facilitator explained to each participant that they would have to perform a secondary task involving mathematical additions. After administration of intravenous anesthetics and when the “patient” had become unconscious, mathematical addition questions were shown on the display. We used simple 2-digit integer additions for the numerical calculations, with the addition questions being shown on the display every 5 seconds, together with an electronic sound signal. While participants were performing endotracheal intubation, we stopped showing the questions because the participants were not looking at the display at this time. When all the questions were answered, the facilitator considered the scenario as being completed. A total of 40 addition questions were shown and their correct answer rate in scenario B was calculated (correct answer rate B). Two faculty raters calculated the key action score in scenario B (key action score B) and correct answer rate B of addition questions.

### 2.3. Scenario C: Combination of Simple Addition and Treatment of Unexpected Arrhythmia

We simulated a situation of induction of general anesthesia in healthy patients, as in scenario A. Further, before the scenario commenced, the facilitator explained to each participant that they would have to perform a secondary mathematical addition task, as in scenario B. After administration of the intravenous anesthetics and when the “patient” was no longer conscious, addition questions were shown on the display, as in scenario B. However, before the participants tried to perform endotracheal intubation, the simulated patient developed paroxysmal supraventricular tachycardia (PSVT), which the participant had not been previously informed about and which was untreatable with any procedure or any drug. The operator controlled the simulated patient's heart rate (180 bpm) and blood pressure (75–85/35–45 mmHg). Two faculty raters calculated the key action score in scenario C (key action score C) and correct answer rate of addition questions in scenario C (correct answer rate C) during this part of the study.

Estimation of scores of key actions 1 and 4 is as follows: if these actions were successfully performed, the actions were assigned a score of 1 each. If participants could not perform these actions, their score was 0.

Estimation of scores of key actions 2 and 3 is as follows: if the drugs were administered without delay, the tasks were assigned a score of 1. If administration of the drugs was delayed remarkably compared to baseline or participants did not administer the drugs at all, they received a score of 0. The total score of key actions ranged from 0 to 4.

In addition, the two faculty raters counted any treatment of PSVT by each participant, such as medication, Valsalva maneuver, or cardioversion.

All the scenarios were recorded on video that was later evaluated by the two faculty raters for score assessment or counting the number of treatments of PSVT. The performance of the anesthesiologists was evaluated in terms of the collective answer rate of numerical calculations and the scores of key actions compared with scenario A. Welch's *t*-test was used for comparisons of correct answer rate and number of treatments for PSVT between the groups and scenarios. A statistically significant difference was defined as *p* < 0.05. The 95% confidence intervals for the difference of 2 medians were also calculated. Mann-Whitney *U* test was used for comparisons of key action scores. A statistically significant difference was defined as *p* < 0.05.

## 3. Results

**Figure 2:**
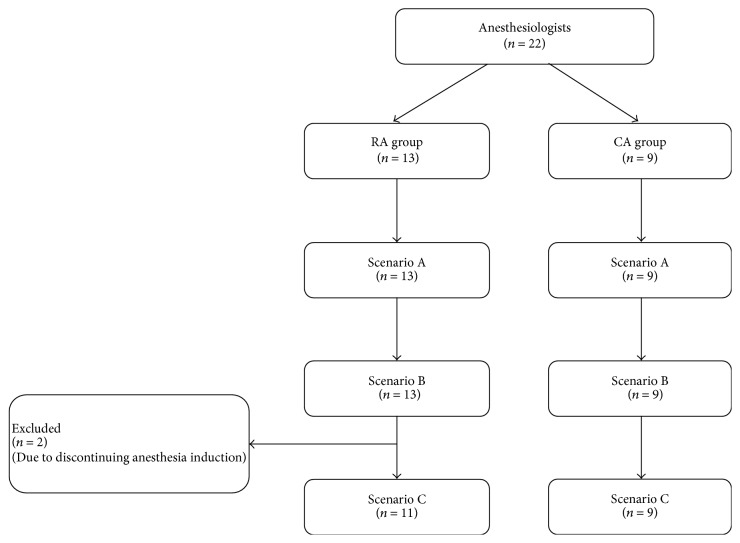
Flow diagram of inclusion in the study. Two anesthesiology residents were excluded as they discontinued anesthesia induction. RA: anesthesiology resident. CA: certified anesthesiologist.

All 22 participants completed scenarios A and B. However, two of the participants did not continue the induction of anesthesia as part of their attempt to treat the PSVT during scenario C, as they believed that the PSVT resulted from anesthetic drug administration and, hence, discontinuation of anesthesia induction would be preferable for patient safety. We excluded these participants from analysis of scenario C (Figure 2).

### 3.1. Scenario A

We simulated standard general anesthesia induction with inhalation anesthetics in scenario A.

We confirmed that all participants had standard anesthetic skills (e.g., mask ventilation and tracheal intubation) and did not have any problems to use the mannequin.

### 3.2. Scenario B

**Figure 3:**
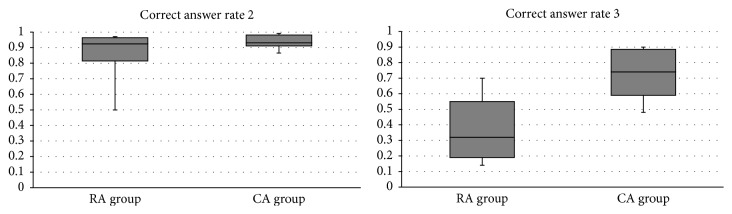
Correct answer rate of numerical calculation in scenarios 2 and 3. Comparison of the correct answer rates for the 40 numerical calculations between RA and CA groups in scenarios B and C. Data are expressed as total correct answer/40. RA: anesthesiology resident. CA: certified anesthesiologist.

In scenario B, the rate of correct responses to the mathematical questions was not significantly different between the two groups (RA: 0.853 ± 0.162 versus CA: 0.945 ± 0.046, *p* = 0.07, 95% CI −0.193 to 0.010) (Figure 3). The total score of key actions compared with scenario A was also similar between the two groups (RA: 3.6 ± 0.65 versus CA: 4.0 ± 0.00, *p* = 0.07).

### 3.3. Scenario C

**Figure 4:**
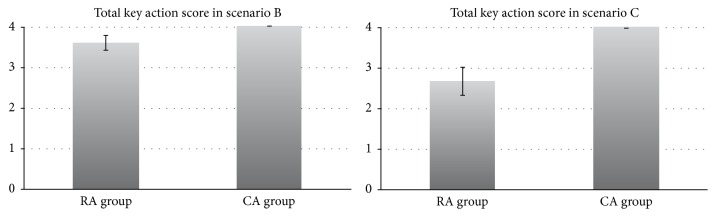
Comparison of total key action scores between RA and CA groups in scenarios B and C. RA: anesthesiology resident. CA: certified anesthesiologist.

The rate of correct answers to the mathematical questions in scenario C was significantly higher in the CA group than the RA group (RA: 0.370 ± 0.050 versus CA: 0.736 ± 0.051, *p* = 0.0007, 95% CI −0.518 to −0.215) (Figure 4).

The total score of key actions was also significantly higher in the CA group than the RA group (RA: 2.7 ± 1.3 versus CA: 4.0 ± 0.00, *p* = 0.005). The score of key action 2 was significantly higher in the CA group (*p* < 0.01). The score of key action 3 in scenario C compared with scenario A tended to be higher in the CA group, although the difference between the two groups was not significant (*p* = 0.04).

The rate of correct answers to the mathematical questions and the score of key actions compared with scenario A in both groups decreased in scenario C as compared to scenario B. In group RA, the decrease in the correct answer rate fell remarkably from 0.853 to 0.370 in scenario C versus scenario B, compared with the decrease in group CA from 0.945 to 0.736.

The number of treatments for PSVT was not significantly different between the two groups (RA: 3.85 ± 2.19 versus CA: 3.33 ± 1.58, *p* = 0.266, 95% CI −1.1658 to 2.1915).

## 4. Discussion

In this study, the need to simultaneously perform the primary task of general anesthesia induction and a simple secondary task did not significantly influence the performances of both the novice and experienced anesthesiologists in scenario B. For anesthesiologists, induction of general anesthesia in healthy patients is not difficult as it is a part of their daily clinical practice. If, however, the primary task had been close to or exceeds the operator's maximal capacity, such as insertion of a central venous catheter or performance of a peripheral nerve block, the effects of interruption of this primary task by a simple secondary task, on both the primary and secondary tasks, may have been greater.

Previous studies showed the influence of a secondary task during general clinical anesthesia [17, 18]. In these studies, the response to the secondary task, which involved responding to a vibrating mobile phone during clinical general anesthesia practice (primary task), occurred later for the novice anesthesiologists as compared with the experienced anesthesiologists [12, 13]. In the previous study, because the secondary tasks were randomly given to the participants while they administered general anesthesia in the clinical setting, the primary workloads might have been variable. Hence, the previous researchers could not evaluate the interaction between primary and secondary tasks. In our simulation study, since we fixed the workloads of primary and secondary tasks, we tried to evaluate the influence of interactive effect between the primary and secondary tasks. In addition, we used calculations as visual stimulus unlike previous studies.

In scenario C in this study, we tried to evaluate the interactive effect between the primary and secondary tasks in a more serious situation. The score of the key actions and the correct answer rate in this setting decreased in both groups as compared with scenario B. However, the novice anesthesiologists tended to show a greater decrease in their scores as compared to the experienced anesthesiologists.

Unexpected arrhythmia during induction of anesthesia is a very stressful situation for anesthesiologists. However, in this study, because the simulated patient's blood pressure remained stable during PSVT, he did not immediately need cardioversion or any medication [19, 20]; the most experienced anesthesiologists remained calm and did not treat PSVT in a hurry or discontinue their anesthetic procedures in this study. On the other hand, less experienced anesthesiologists struggled to treat PSVT and their mental workload was close to their capacity.

In a serious clinical situation, anesthesiologists might not be able to adequately perform both the primary and secondary tasks. These results mean that mental workload of the primary task is close to the participant's maximal capacity. This tendency is more apparent in young anesthesiologists.
